# Berotralstat for hereditary angioedema with C1 inhibitor deficiency: a practical guide for clinicians

**DOI:** 10.3389/fimmu.2024.1442671

**Published:** 2024-10-08

**Authors:** Adil Adatia, Markus Magerl

**Affiliations:** ^1^ Division of Pulmonary Medicine, Department of Medicine, University of Alberta, Edmonton, AB, Canada; ^2^ Institute of Allergology, Charité - Universitätsmedizin Berlin, Freie Universität Berlin and Humboldt-Universität zu Berlin, Berlin, Germany; ^3^ Immunology and Allergology, Fraunhofer Institute for Translational Medicine and Pharmacology ITMP, Berlin, Germany

**Keywords:** hereditary angioedema, C1 inhibitor, kallikrein, berotralstat, immunodeficiency

## Introduction

Hereditary angioedema due to C1 inhibitor deficiency (HAE-C1INH) is a rare congenital disorder that causes episodic swelling. Decreased or dysfunctional C1INH, the key inhibitor of the kinin-kallikrein system, leads to increased plasma kallikrein activity and the resultant excessive bradykinin causes angioedema (1).

The morbidity and decreased quality of life of HAE-C1INH patients has motivated the increasing use of long-term prophylaxis (LTP) to prevent swelling attacks, when modern LTP agents became available a few years ago. The oral kallikrein antagonist berotralstat was approved in the United States and European Union in 2020 and 2021, respectively. The 2021 international HAE-C1INH guideline recommends berotralstat as a first-line therapy alongside plasma-derived C1 inhibitor (pd-C1INH) and lanadelumab (2).

Herein, we present a practical guide to aid the clinician in the optimal use of berotralstat in HAE-C1INH based on available data and the experience at the Angioedema Centers of Reference and Excellence (3) in Berlin, Germany and Edmonton, Canada.

## Drug pharmacology

Berotralstat is an oral small molecule that specifically inhibits plasma kallikrein. It is administered once daily at a dose of 150 mg. In the United States, a 110 mg dose is also available. It achieves a steady state plasma concentration in 6-12 days and has an elimination half-life of 93 hours (4). Administration with food does not impact the maximum serum concentration or total exposure of the medication (4).

## Clinical efficacy

The efficacy of berotralstat was established in a double-blind, parallel-group study of 24 weeks duration (APEX-2). There were n=121 subjects randomized 1:1:1 to placebo, berotralstat 150 mg daily, or berotralstat 110 mg daily, and there was a 44% and 30% reduction in attack frequency/month in the treatment versus placebo arm at the 150 and 110 mg doses, respectively (5). The subsequent extension studies showed persistent and perhaps increased efficacy over time, with the 150 mg arm achieving a 79% reduction in attack frequency compared to baseline (6, 7) after 6 months (n=21). Real-world evidence studies have confirmed these results in various countries (8–10).

## Side effects and monitoring

Berotralstat was generally well tolerated at both doses. There was one severe adverse event (SAE) in the 110-mg berotralstat and three SAEs in the placebo arm, none of which were classified as drug related (5). The most frequent side effects observed in APEX-2 were gastrointestinal (GI) symptoms (*e.g.*, abdominal discomfort, diarrhea). These were seen in 50% (20/40) of subjects receiving berotralstat 150 mg *versus* 36% (14/39) of subjects receiving placebo but tended to resolve over time. The GI symptoms can be mitigated by taking the medication with the largest meal of the day. Additionally, the graded introduction of berotralstat using the Berlin protocol, which involves starting with 1 tablet (150 mg) every third day and then increasing dosing frequency as tolerated to 1 tablet every other day and finally to 1 tablet daily, can improve tolerability (11). Some clinicians worry that GI upset may be misattributed to abdominal angioedema and result in inappropriate use of on-demand therapy. But, in our experience, patients (including those who have and have not had previous abdominal episodes) are generally able to differentiate between these when given anticipatory guidance. Before initiating therapy with berotralstat, patients must be informed about the possibility of gastrointestinal side effects, but care should be taken not to overemphasize this point in order to avoid nocebo effects. Additionally, headache was seen in 10.0% (4/40) of patients in the 150 mg arm of APEX-2 but also in 5.1% (2/39) of patients in the placebo arm. In our experience, it has not been a patient concern.

QT interval prolongation was observed with berotralstat at doses 3-fold greater than that used clinically. At the approved dose of 150 mg daily berotralstat does not cause significant QT interval prolongation and electrocardiographic monitoring is not required. In APeX-2, one patient discontinued therapy due to elevated liver enzymes in the context of prior therapy with attenuated androgens. However, no regular laboratory monitoring is needed according to the drug monograph (4) and we have not observed elevated liver enzymes in our patients.

## Drug-drug interactions

Berotralstat is metabolized in the liver by CYP2D6 and CYP3A4 and hence may increase the plasma concentration of concomitant medications also metabolized by these enzymes (**Table 1**) (4). In some instances, reducing the dose of an interacting medication may be helpful. For example, one author has combined amlodipine with berotralstat and reduced the dose of the former (amlodipine 10 mg to 5 mg daily) without any loss of blood pressure control. Patients using a statin also metabolized by CYP3A4 (*e.g.*, atorvastatin) can be switched to rosuvastatin, which is not a major CYP3A4 substrate (12) and thus does not interact with berotralstat. Given the potential increased risk of bleeding in patients taking the direct oral anticoagulants rivaroxaban and apixaban, switching patients to edoxaban or dabigatran prior to starting berotralstat is advisable.

**Table 1:** Selection of commonly used medications that may interact with berotralstat.

Drug (Interaction Risk)	Recommendation
Calcium channel blockers	
Verapamil (D)	Consider therapy modification
Amlodipine (C)	Monitor blood pressure, consider dose reduction
Nifedipine (C)	Monitor blood pressure, consider dose reduction
Diltiazem (C)	Monitor blood pressure, consider dose reduction
Statins	
Atorvastatin (C)	Monitor for side effects (*e.g.*, myalgias), consider switch to rosuvastatin
Simvastatin (C)	Monitor for side effects (*e.g.*, myalgias), consider switch to rosuvastatin
Lovastatin (C)	Monitor for side effects (*e.g.*, myalgias), consider switch to rosuvastatin
Anticoagulants	
Apixaban (C)	Consider switch to edoxaban or dabigatran
Rivaroxaban (C)	Consider switch to edoxaban or dabigatran
Sedative Hypnotics	
Alprazolam (D)	Consider therapy modification
Zopiclone (C)	Consider dose reduction and monitor for side effects (*e.g.*, somnolence)
Diazepam (C)	Consider dose reduction and monitor for side effects (*e.g.*, somnolence)
Opioids	
Fentanyl (D)	Consider therapy modification
Codeine (C)	Consider dose reduction and monitor for side effects (*e.g.*, somnolence, constipation)
Tramadol (C)	Consider dose reduction and monitor for side effects (*e.g.*, somnolence, constipation)

Interactions were determined using the Lexicomp Drug Interactions Tool (13), which grades interactions as no action required **(B)**, monitor therapy **(C)**, consider therapy modification **(D)**, and avoid combination (X). This list is not exhaustive.

Berotralstat is also a P-glycoprotein (P-gp) substrate. Thus P-gp inducers (*e.g.*, rifampin, St. John’s wort) may decrease the serum concentration of berotralstat and should be avoided. P-gp inhibitors (*e.g.*, carvedilol) could increase the plasma concentration of berotralstat and therefore the lower 110 mg dose of berotralstat should be used in such patients, if available.

## Switching LTP therapies

There is a paucity of data to guide clinicians on how to transition a patient from one LTP treatment to another. When switching to berotralstat, the main issues of concern are possible breakthrough attacks during the transition period, heightened patient anxiety regarding the possibility of breakthrough attacks, and premature discontinuation of an effective treatment due to breakthrough attacks prior to the new drug taking effect. The possibility of breakthrough attacks is likely greatest when transitioning from pd-C1INH therapy due to its half-life (69 hours for subcutaneous pd-C1INH [Haegarda^®^/Berinert 2000 and 3000^®^] and 56 hours for intravenous pd-C1INH [Cinryze^®^]). Since berotralstat achieves a plasma steady state within 2 weeks, it is reasonable to overlap it with pd-C1INH by up to 4 weeks (8). This could be extended when the Berlin protocol of graded introduction is used to ensure that the first weeks of daily berotralstat are covered by pd-C1INH.

The half-life of lanadelumab is 2-3 weeks, so if daily berotralstat is started immediately after the last dose of lanadelumab, further overlap is unlikely to be needed. Though the mechanism of action of both lanadelumab and berotralstat is plasma kallikrein inhibition, they bind to kallikrein at different locations [lanadelumab binds to the surface of the enzyme and obstructs substrate access to the active site (14) whereas berotralstat binds deep within the active site (15)], and patients hence may have different responses to each drug. Failure to respond to one should, therefore, not preclude the use of the other.

When transitioning from attenuated androgen therapy to berotralstat, caution is required given the risk of liver enzyme elevation in a minority of patients. This was seen in those who stopped androgens <14 days before starting berotralstat and may be due to abrupt discontinuation of androgens and/or latent hepatoxic effects of androgens that emerge if berotralstat is started too soon after androgen discontinuation (16). We thus suggest a gradual tapering of androgen therapy and a washout period of 14 days prior to starting berotralstat. The washout phase of androgens and the time to steady state of berotralstat can be covered with temporary pd-C1INH until the full efficacy of berotralstat has been reached. In jurisdictions where this is not feasible due to logistical or cost considerations, co-administration of berotralstat and androgen therapy whilst gradually reducing the androgen dose (and monitoring liver enzymes) could be considered as part of a shared decision-making process.

In clinical practice, scenarios other than just the smoothest possible transition from one long-term prophylaxis to another are also conceivable. The interruption of long-term prophylaxis (and temporary on-demand therapy alone) can be used to re-establish the indication for continuing long-term prophylaxis with another therapy. Ultimately, switching long-term prophylaxis therapies is a highly individualized decision in which the patient’s values and preferences should play an important role.

## Conclusion

Berotralstat is an effective treatment and provides patients with an oral LTP option. GI side effects occur in a minority of patients, can be mitigated by graded introduction, and tend to resolve over time. Berotralstat may increase the plasma concentrations of certain concomitant medications, and dose reductions of those medicines may be needed. When switching to berotralstat from pd-C1INH, a treatment overlap period could help prevent breakthrough angioedema episodes during the transition period. Prospective studies to help establish optimal transition protocols are warranted.
